# Ablation of Protein Kinase CK2β in Skeletal Muscle Fibers Interferes with Their Oxidative Capacity

**DOI:** 10.3390/ph10010013

**Published:** 2017-01-19

**Authors:** Nane Eiber, Luca Simeone, Said Hashemolhosseini

**Affiliations:** Institute of Biochemistry, Friedrich-Alexander University of Erlangen-Nuremberg, Fahrstrasse 17, 91054 Erlangen, Germany; nane.eib@web.de (N.E.); simeoneluca@gmail.com (L.S.)

**Keywords:** protein kinase CK2, skeletal muscle, C2C12, myopathy

## Abstract

The tetrameric protein kinase CK2 was identified playing a role at neuromuscular junctions by studying CK2β-deficient muscle fibers in mice, and in cultured immortalized C2C12 muscle cells after individual knockdown of CK2α and CK2β subunits. In muscle cells, CK2 activity appeared to be at least required for regular aggregation of nicotinic acetylcholine receptors, which serves as a hallmark for the presence of a postsynaptic apparatus. Here, we set out to determine whether any other feature accompanies CK2β-deficient muscle fibers. Hind limb muscles gastrocnemius, plantaris, and soleus of adult wildtype and CK2β-deficient mice were dissected, cross-sectioned, and stained histochemically by Gomori trichrome and for nicotinamide adenine dinucleotide (NADH) dehydrogenase and succinate dehydrogenase (SDH) enzymatic activities. A reduction of oxidative enzymatic activity was determined for CK2β-deficient muscle fibers in comparison with wildtype controls. Importantly, the CK2β-deficient fibers, muscle fibers that typically exhibit high NADH dehydrogenase and SDH activities, like slow-type fibers, showed a marked reduction in these activities. Altogether, our data indicate additional impairments in the absence of CK2β in skeletal muscle fibers, pointing to an eventual mitochondrial myopathy.

## 1. Introduction

CK2 is a highly conserved, ubiquitously expressed serine/threonine kinase present in all eukaryotes [1,2]. It is involved in many biological processes, such as proliferation, apoptosis, differentiation, and tumorigenesis. The CK2 holoenzyme consists of a tetramer of two catalytic (α/α′) and two regulatory (β) subunits. Ablation studies have demonstrated the inability of CK2α to compensate for the loss of CK2α′ during mouse spermatogenesis [3,4], suggesting functional specialization. In mice, disruption of the gene encoding the CK2β subunit is lethal at a very early embryonic stage [5]. The precise mode of regulation of CK2 activity is poorly defined; i.e., as to whether CK2 is constitutively active or modulated in response to stimuli [1]. Recently, the involvement of CK2 in Wnt signaling, namely the canonical Wnt/β-catenin signaling pathway, has been reported [6,7]. Non-canonical Wnt signaling, like the planar-cell polarity pathway, is implicated in post-synaptic cytoskeletal reorganization. Members of Wnt signaling pathways, such as β-catenin, disheveled, and the tumor suppressor protein adenomatous polyposis coli, directly associate with proteins assembling the post-synaptic apparatus [8,9,10]. Canonical Wnt signaling is even active in adult skeletal muscle fibers [11]. Previously, CK2 was identified in mammalian skeletal muscle cells phosphorylating glycogen synthase from rabbit skeletal muscle [12]. Later, CK2 was reported being linked to the aggregation of nicotinic acetylcholine receptors and interacting with several components of the postsynaptic machinery, regardless whether in cultured immortalized C2C12 cells or in mice [13,14]. CK2β was shown to strongly interact with the phosphorylated intracellular domain of the muscle-specific receptor tyrosine kinase MuSK at the neuromuscular junction (NMJ) [13]. This interaction requires the entire intracellular MuSK domain with the exception of the C-terminal PDZ-binding motif, as well as the positive regulatory domain of CK2β [13]. Further, phosphorylation of serine residues within the kinase insert domain of MuSK by CK2 was demonstrated [13]. Importantly, phosphorylation of MuSK by CK2 prevents fragmentation of the NMJs [13]. Generation and characterization of myotube-specific CK2β knockout mice corroborated in vitro data and showed loss of muscle grip strength in an age-dependent fashion [13]. Later, at the NMJ CK2 was shown to additionally interact with the α and β subunits of the nicotinic acetylcholine receptors (AChR), disheveled, and strongly with Rapsyn, Rac1, 14-3-3γ, and Dok-7 [14]. It turned out that CK2 phosphorylates 14-3-3γ and Dok-7, but not Rapsyn or Rac1, at several serine residues [14]. Importantly, phosphomimetic Dok-7 mutants aggregate AChRs in C2C12 myotubes with a significantly higher frequency than wildtype Dok-7 [14]. In another report, 34 muscle biopsies of human patients with different muscle pathologies were analyzed regarding CK2 transcript amount and enzymatic activity. Interestingly, holoenzyme CK2 kinase activity was highly variable in limb girdle muscular dystrophy (LGMD) patients and appeared to be lower in mitochondrial myopathy patients [15].

Here, we set out to determine whether different histochemical stainings of hind limb muscle cross-sections of CK2β-deficient mice can enable us to gain more insight into potential extra-synaptic impairments of their skeletal muscle fibers. In comparison with wildtype controls, accumulated aggregates were detectable at the subsarcolemmal membrane of CK2β-deficient skeletal muscle fibers and accompanied by a lack of oxidative enzymatic activities of nicotinamide adenine dinucleotide (NADH) dehydrogenase and succinate dehydrogenase (SDH).

## 2. Results

**Figure 1:**
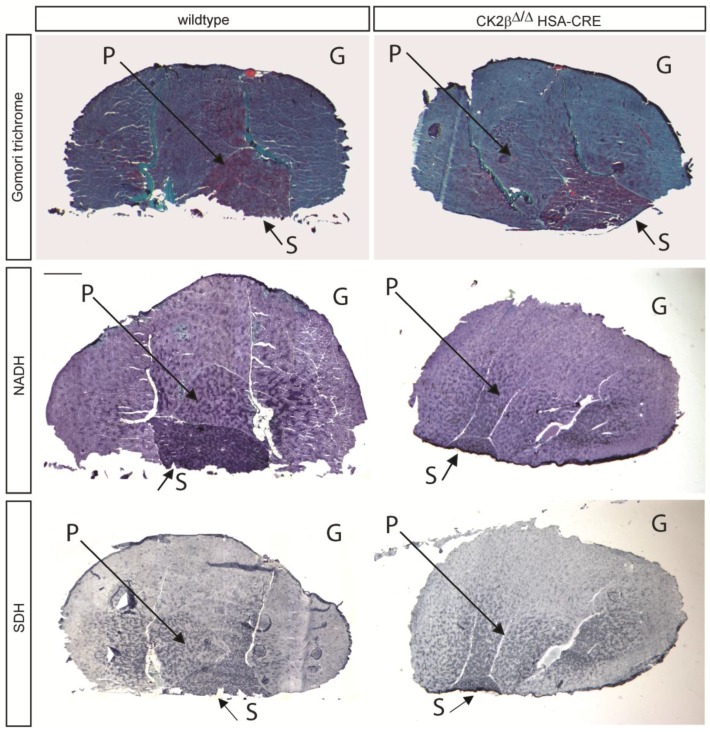
Typical images of full size hind limb cross sections after application of different types of histochemical stainings show gastrocnemius (G), plantaris (P), and soleus (S) of wildtype and CK2β-deficient (CK2^Δ/Δ^ HSA-Cre) muscles of mice. Histochemical stainings by Gomori trichrome and for detection of NADH dehydrogenase and SDH enzyme activities are indicated.

**Figure 2:**
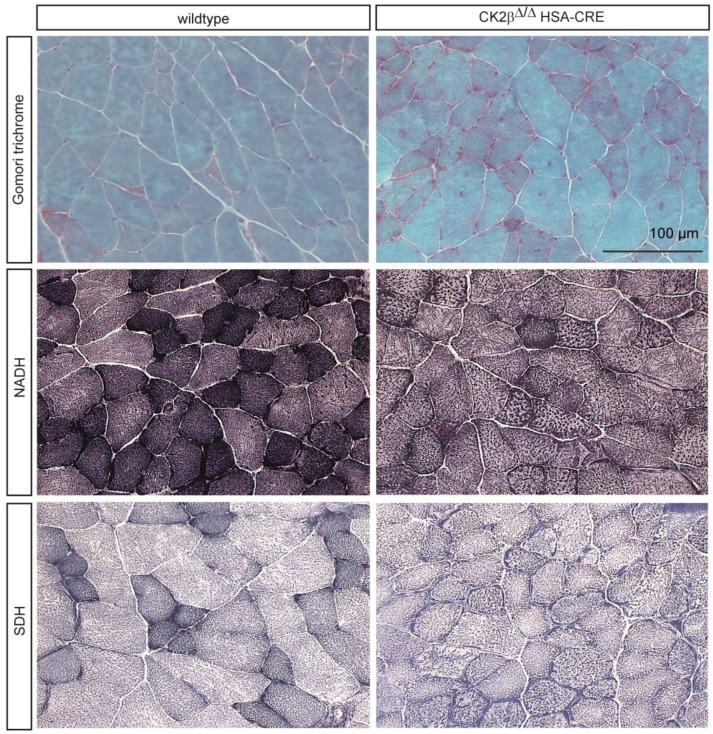
Typical images of hind limb cross sections after different histochemical stainings show plantaris muscles of wildtype and CK2β-deficient mice at high resolution. Note that mitochondria appear accumulated at the subsarcolemmal membrane of CK2β-deficient muscle fibers (red-colored in Gomori trichrome-stained muscle sections), while after NADH dehydrogenase and SDH stainings dark-colored slow type muscle fibers in wildtype cross sections are less dark-colored in CK2β-deficient muscle fibers. Further, in comparison with wildtype muscles fiber diameters are significantly reduced on mutant muscle cross sections. The scale bar shown is representative for all images of muscle cross-sections in this figure.

**Figure 3:**
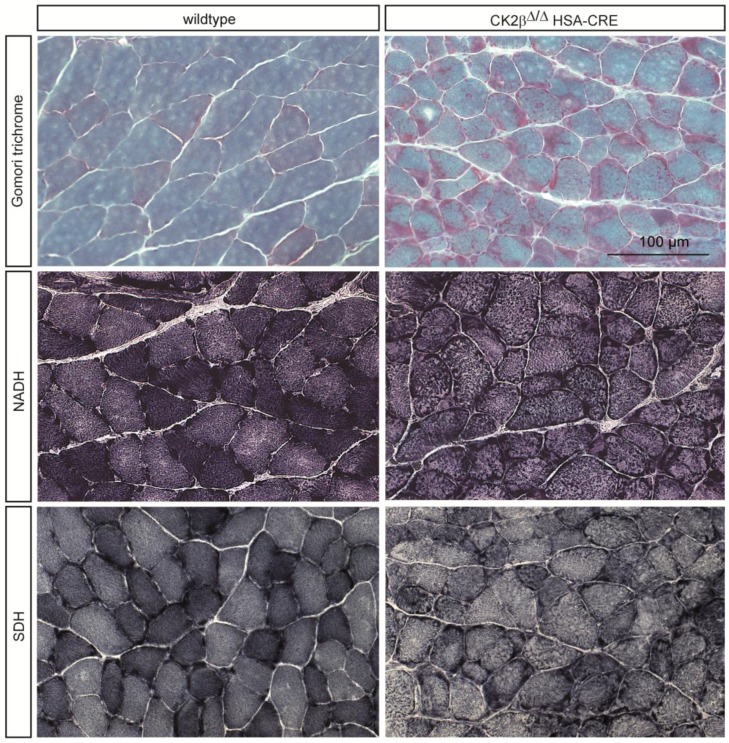
High-resolution images of hind limb cross sections after different histochemical stainings show soleus muscles of wildtype and CK2β-deficient mice. Note that, in comparison with wildtype controls, cross sections of CK2β-deficient muscle fibers are of more granular appearance after NADH dehydrogenase and SDH staining, and there are a significant number of fibers with lower diameter. The scale bar shown is representative for all images of muscle cross-sections.

CK2 is ubiquitously expressed and was reported to be further accumulated at the postsynaptic apparatus of skeletal muscle fibers [13,14]. We wondered about the subcellular localization of extra-synaptic CK2 within muscle fibers. After transfection of protein fusions of individual CK2 subunits with GFP into primary cultured muscle cells and their differentiation to myotubes, the GFP fluorophore was detected all along the myotubes, indicating that CK2 might be also necessary at extra-synaptic sites (data not shown). To look for extra-synaptic changes, mutant hind limb muscle fiber cryosections of adult (>6–8 months) wildtype and mutant mice were stained with hematoxylin and eosin. Gastrocnemius, plantaris, and soleus muscle cross sections possessed fibers of smaller and variable diameters, containing a significantly higher number of central nuclei and partly subsarcolemmal aggregations (data not shown). We set out to determine whether other types of histochemical stainings might elucidate potential changes comparing wildtype and CK2β-deficient muscles. We decided to choose the hind limb muscles, gastrocnemius, plantaris, and soleus, which represent examples of both glycolytic and oxidative skeletal muscles. While gastrocnemius is mostly composed of glycolytic fibers, plantaris contains some oxidative fibers. The soleus muscle in mice is mainly composed of glycolytic and oxidative fibers in a similar ratio. Low-resolution images of different histochemical stainings demonstrated subsarcolemmal aggregates on cryosections from mutant, but not wildtype muscles (Figure 1). Further, a high number of tiny little speckles were visible on mutant sections and not on the wild types, especially at high-resolution (Figure 1, Figure 2 and Figure 3). We proceeded to examine histochemical stainings of the hind limb muscles by high-resolution microscopy and densitometrical quantifications of staining patterns (Figure 2 and Figure 3).

### 2.1. Gomori Trichrome Staining Revealed Impairments in CK2β-Deficient Muscle Fibers

Gomori trichrome is a staining procedure that combines the plasma and connective fiber stain in a phosphotungstic acid solution to which glacial acetic acid has been added [16]. It is commonly used to identify potential metabolic impairments in human muscle pathologies, likely indicating mitochondrial myopathies. Skeletal muscle fibers containing structurally abnormal mitochondria below the sarcolemmal membrane and within the fiber itself that stain red with Gomori trichrome staining are occasionally seen in mitochondrial myopathies and in other myopathy disorders. Cross sections of plantaris and soleus muscle from adult wildtype and CK2β-deficient mice were stained with Gomori trichrome and imaged by high-resolution microscopy (Figure 2 and Figure 3). Interestingly, muscles from CK2β-deficient mice in comparison with wildtype controls contained red-colored subsarcolemmal aggregates within their muscle fibers (Figure 2 and Figure 3). Most likely, these aggregates indicate abnormal mitochondria which accumulated at the subsarcolemmal membrane (Figure 2 and Figure 3).

### 2.2. Nicotinamide Adenine Dinucleotide (NADH) Dehydrogenase Staining Detected a Diminished Enzymatic Activity in CK2β-Deficient Muscle Fibers

*NADH* tetrazolium reductases are flavoprotein enzymes that have the property of transferring hydrogen from a reduced nicotinamide adenine dinucleotide (NADH) to various dyes. Usually, tetrazolium compounds function as the hydrogen acceptor when these reductases are being demonstrated histochemically, and the product of the reduction is the water-insoluble purple-blue formazan pigment marking the site of enzyme activity [17]. Accordingly, this staining detects the activity of the enzyme NADH dehydrogenase, which is also called NADH/coenzyme Q-Oxidoreductase; or briefly NADH reductase. Here, we will use the term NADH dehydrogenase or NADH dehydrogenase staining. We continued investigating cross sections of plantaris and soleus muscle from adult wildtype and CK2β-deficient mice by NADH dehydrogenase staining (Figure 2 and Figure 3). By Gomori trichrome staining, we observed that muscles from CK2β-deficient mice appear to be accompanied by abnormal mitochondria (Figure 2 and Figure 3). We wondered whether changes in the oxidative capacity of CK2β-deficient skeletal muscle fibers were reflected in NADH dehydrogenase staining. We detected a significant number of dark-colored fibers in wildtype muscle cross sections, pointing to slow fiber types known to contain a high number of mitochondria (Figure 2 and Figure 3). In comparison, CK2β-deficient skeletal muscle fibers did not possess these dark-colored NADH dehydrogenase stained fibers. Instead, CK2β-deficient muscles contained fibers which were only slightly more colored compared to neighboring fast fibers (Figure 2 and Figure 3). The loss of color intensity of slow type fibers was densitometrically examined and compared between wildtype and CK2β-deficient skeletal muscle fibers (Figure 2 and Figure 3). CK2β-deficient slow type muscle fibers were 0.74-fold less dark-colored in plantaris and 0.76-fold less dark-colored in soleus muscles in comparison with wildtype controls (Table 1).

### 2.3. Succinate Dehydrogenase Activity Is Reduced in CK2β-Deficient Muscle Fibers

Succinic dehydrogenase (SDH) catalyzes the oxidation of succinic acid to fumaric acid. The histochemical activity of this enzyme is detectable as a change in color of muscle fibers after incubation of muscle tissue sections with a succinate substrate in the presence of a tetrazolium compound, a water-soluble compound employed in histochemistry as a redox indicator [17]. The rest of this staining chemistry is comparable to the NADH dehydrogenase staining procedure [17]. Cross sections of adult muscles from wildtype and CK2β-deficient mice were made and stained to reveal SDH activity and determine oxidative capacity of respective skeletal muscle fibers. Importantly, slow fiber types of CK2β-deficient muscles were significantly less intensively dark-colored compared to slow fiber types of wildtype muscles. The change in oxidative capacity of slow type fibers of CK2β-deficient muscles was quantified densitometrically and turned out to be 1.37-fold higher in wildtype, in comparison with CK2β-deficient soleus, and 1.69-fold in comparison with CK2β-deficient plantaris muscle fibers (Table 1).

## 3. Discussion

Up to now, protein kinase CK2 was shown in muscle cells most likely responsible for the maintenance, rather than the formation, of NMJs [13,14]. Interestingly, analyzing CK2 transcript distribution and enzyme activity in human skeletal muscle biopsies from patients suffering either from LGMD of unknown classification, mitochondrial myopathy, or neurogenic muscular atrophy, and normal controls, no significant changes were observed [15]. However, a high variation of CK2 activity was detected in biopsies from LGMD patients [15]. In the case of the mitochondrial myopathy patients, in three out of four biopsies, a decrease of CK2 activity was detected [15]. Here, we examined hind limb muscle cross sections of wildtype and CK2β-deficient mice and looked for additional extra-synaptic impairments by different type of histochemical stainings. By regular hematoxylin and eosin staining, we detected smaller and variable fiber diameters and central nuclei on CK2β-deficient muscle cross sections in comparison with wildtype controls (data not shown). These findings encouraged us to investigate in more detail potential changes between CK2β-deficient and wildtype muscles by applying Gomori trichrome staining and stainings that allow for the measurement of the oxidative capacity of CK2β-deficient muscle fibers by detecting the activities of NADH dehydrogenase and SDH (Figure 2 and Figure 3). It turned out that, by Gomori trichrome, NADH dehydrogenase, and SDH stainings (Figure 2 and Figure 3), mutant fibers were on average reduced in diameter, as detected by hematoxylin and eosin stainings (data not shown). Importantly, Gomori trichrome staining detected accumulations at the subsarcolemmal membrane, most likely pointing to mitochondria and indicating changes in the oxidative capacity of the CK2-deficient skeletal muscle fibers (Figure 1, Figure 2 and Figure 3). During development, several weeks after birth muscle fibers gradually mature into adult muscle fibers. Slow type fibers are known to possess more mitochondria and a higher oxidative capacity. They become dark-colored after NADH dehydrogenase and SDH staining. In this study, we investigated gastrocnemius, plantaris, and soleus muscles by histochemical stainings and detected the oxidative fibers of CK2β-deficient muscle fibers less intensely stained in comparison with wildtype controls (Figure 1, Figure 2 and Figure 3). These less colored fibers in CK2β-deficient muscles appear to be the remains of previously dark-colored oxidative fibers (Figure 2 and Figure 3).

Our data indicate a potential role of the protein kinase CK2 regarding oxidative metabolism in adult skeletal muscle fibers. Further experiments are required to identify the molecular mechanism and understand the potential impact of CK2 regarding human myopathies.

## 4. Materials and Methods

### 4.1. Mice Mating and Genotyping

Mice mating and genotyping were performed as described previously [5,13,18]. Mouse experiments were performed in accordance with animal welfare laws and approved by the responsible local committees (animal protection officer, Sachgebiet Tierschutzangelegenheiten, FAU Erlangen-Nuremberg, AZ: I/39/EE006 and TS-07/11) and government bodies (Regierung von Unterfranken). Mice were housed in cages that were maintained in a room with temperature 22 ± 1 °C and relative humidity 50%–60% on a 12 h light/dark cycle. Water and food were provided ad libitum.

### 4.2. Dissecting of Skeletal Muscles and Tissue Sections

Hind limb muscles were dissected and then quick-frozen in prechilled isopentane as described [13]. Muscles were cryotome-sectioned (10 μm). Cryotome sections were used for histochemical stainings. Sections were embedded in DPX or mowiol (Sigma-Aldrich Chemie GmbH, München, Germany) as earlier described [11,19].

### 4.3. Histochemical Stainings, Immunohistochemistry, Imaging, and Data Analysis

Modified Gomori trichrome staining was perfomed by incubation of muscle sections in Shandon hematoxylin (Fisher Scientific GmbH, Schwerte, Germany) for 15 min, followed by a 15 min wash in tap water and 45 min incubation in Gomori solution (Sigma-Aldrich Chemie GmbH), washed in tap water, and incubated in 100% ethanol.

For nicotinamide adenine dinucleotide (NADH) dehydrogenase staining, sections were incubated for 30 min at 37 °C in a solution containing NBT (Sigma-Aldrich Chemie GmbH), 50 mM Tris/HCl, and NADH (Sigma-Aldrich Chemie GmbH), then washed in distilled water.

For succinate dehydrogenase (SDH) staining sections were incubated for 45 min at 37 °C in a solution containing Tris 0.2 M pH 7.2, cobalt (II)-chloride, MTT (methylthiazolyldiphenyl-tetrazolium bromide), and NADH. Afterwards, sections were incubated for 30 min in 4% PFA and washed in H_2_O.

Stained cryosections were analyzed and documented using a Zeiss Axio Examiner Z1 microscope equipped with an AxioCam MRm camera and ZEISS AxioVision Release 4.8 (Carl Zeiss MicroImaging, Göttingen, Germany). Densitometric quantifications were done using ImageJ [20].

### 4.4. Statistical Analysis

Data are presented as the mean values, and the error bars indicate ± standard deviation. The number of biological replicates per experimental variable (*n*) is usually *n* > 5 or as indicated in the figure legends. The significance was calculated by an unpaired two-tailed *t* test, or as indicated by the figure legends, and are provided as real *p*-values that are believed to be categorized for different significance levels. *** *p* < 0.001, ** *p* < 0.01, or * *p* < 0.05.

## Figures and Tables

**Table 1:** Densitometry measurement of NADH dehydrogenase and SDH staining intensities in slow type fibers of plantaris and soleus muscle of wildtype and CK2β-deficient mice.

Densitometry	Wildtype	CK2β^∆/∆^ HSA-Cre
**Plantaris**		
NADH dehydrogenase	221.6 ± 1.37, *N* = 43	165.9 ± 2.97, *N* = 46
SDH	186.4 ± 2.24, *N* = 50	109.8 ± 1.88, *N* = 46
**Soleus**		
NADH dehydrogenase	221.4 ± 0.87, *N* = 37	169.5 ± 2.46, *N* = 47
SDH	193.8 ± 1.30, *N* = 35	141.0 ± 2.90, *N* = 42
